# Dividend signaling or dividend smoothing? New empirical evidence from the italian insurance industry after the global financial crisis

**DOI:** 10.1007/s12297-022-00542-3

**Published:** 2022-12-08

**Authors:** Tobias Basse, Sebastian Reddemann, Miguel Rodriguez Gonzalez

**Affiliations:** 1 Norddeutsche Landesbank (NORD/LB), Friedrichswall 10, 30159 Hannover, Germany; 2 Touro College Berlin, Am Rupenhorn 5, 14055 Berlin, Germany; 3 VHV Versicherungen, Constantinstr. 90, 30177 Hannover, Germany; 4 Leibniz Universität Hannover, Otto-Brenner-Str. 7, 30159 Hannover, Germany

**Keywords:** E44, G18, G35, G38

## Abstract

This empirical study examines the dividend policy of insurance companies in Italy after the Global Financial Crisis. There is clear evidence for dividend signaling in this period of time. Moreover, the relationship between stock prices and dividend payments is analyzed in more detail. Additionally, the paper also discusses macroeconomic and regulatory issues that could be of relevance for the dividend policy of the Italian insurance industry. In this context the study exemplarily discusses the possible role of inflation and of regulatory restrictions on dividend payouts in the financial services industry.

## Abstract (de)

Diese empirische Studie untersucht die Dividendenpolitik italienischer Versicherungsunternehmen nach der Globalen Finanzkrise und präsentiert klare Hinweise auf die Umsetzung der Strategie des „Dividend Signalings“ durch die Firmen. Zudem wird der Zusammenhang zwischen Aktienkursen und Dividendenzahlungen detaillierter analysiert. Weiterhin werden makroökonomische und regulatorische Fragestellungen diskutiert, die für die Dividendenpolitik der Firmen von Bedeutung sein könnten. In diesem Kontext wird zum Beispiel auf Inflation und auf regulatorische Vorgaben bezüglich der Höhe von Dividendenausschüttungen eingegangen.

## Introduction

While dividend policy issues certainly are considered to be a very important topic in the field of corporate finance, there is still no clear picture why firms pay dividends and why investors seem to like dividends (see, for example, Frankfurter 1999; and Baker and Weigand 2015). As a consequence, the existence of dividend payments in the “real world” of finance is often described with the expression “puzzling” (see, most importantly, Black 1976; and—more recently—Baker and Powell 2012; as well as Jabbouri and El Attar 2018). There are numerous relevant research efforts which, for example, have been reviewed by Bhattacharyya (2007) and Jabbouri and El Attar (2018). However, examining the literature it has to be concluded that there still is no clear picture (see, for instance, Baker et al. 2002; and Bhattacharyya 2007). As a matter of fact, Baker et al. (2002) have stressed that despite of a voluminous amount of research not all answers to un-puzzle the dividend puzzle have been found yet. They have noted that the dividend policy preferred by the managers of a corporation can differ substantially from one firm to another. Thus, it could make sense to analyze dividend policy issues by focusing on firm specific factors. Nevertheless, there are also opinions that differ from this point of view. In fact, Van Caneghem and Aerts (2011) have argued convincingly that firms that belong to one industry at least to a certain extent seem to imitate the dividend policy of their direct rivals. Thus, it could also be quite informative to, for example, empirically analyze dividend policy issues based on data from a specific sector of the economy. Actually, the question of an industry’s influence on the dividend policy of firms has been investigated for some time now (see, most importantly, Michel 1979; and Baker 1988). While there is some prior work that is of relevance, this approach has gained popularity only recently.

Empirical tests of different explanations for the existence of dividends in the past have focused very strongly on corporations outside the financial sector. But, as we will discuss later, financial firms could show a significantly differing behavior due to their unique regulatory regime. Basse et al. (2011), for instance, have examined the dividend policy of car manufacturers in Germany. Meanwhile there are also papers analyzing data from the financial services industry Recently, Basse et al. (2014) have focused on the dividend policy of European banks. Moreover, there are a number of relevant studies that focus on dividend policy issues in the European insurance industry (see, amongst others, Reddemann et al. 2010; and Basse 2019). Given that dividend reductions or even dividend omissions have been suggested as one measure to strengthen the capital base of financial services firms as a reaction to the problems that could result from macroeconomic crisis events (see, for example, Reddemann et al. 2010; and Jakubik and Teleu 2021) these empirical studies might have a special importance. One of the more recent papers from this sub-set of the literature has examined data from Italian insurance companies (see Basse et al. 2011a). Italy has had to face a number of crisis events in recent years (see, for instance Basse et al. 2012; and Tholl et al. 2020). Thus, further empirical evidence from the Italian insurance industry could be very interesting. Furthermore, Jakubik and Teleu (2021) have stressed that the European Insurance and Occupational Pensions Authority (EIOPA) and the national insurance regulator in Italy have tried to take measures in order to limit the ability of insurance companies to pay dividends.

Consequently, we examine the dividend policy of Italian insurers focusing on the more recent experiences (which means analyzing data from the time period after the Global Financial Crisis) and employ techniques of time series analysis to do so. Generally speaking, our research approach follows Goddard et al. (2006). More specifically, the concept of Granger causality is used here (see Granger 1969; and Granger 2003). Stated even more clearly, we analyze dividend payouts and corporate earnings examining aggregated data from insurance companies in Italy by employing the technique that has been developed by Toda and Yamamoto (1995) to test for Granger causality. Furthermore, we then also analyze the relationship between dividend payouts and stock prices.

The paper is structured as follows. In Sect. 2 dividend policy issues are discussed from the viewpoint of the theory of corporate finance. Sect. 3 then briefly reviews the relevant literature focusing on the insurance industry. After considering some more general questions, the existing empirical literature that analyzes the dividend policy of the European insurance industry is discussed here in some detail. Sect. 4 examines the recent crisis events in Italy that possibly could have an influence on the dividend policy of the financial services firms in this country. Sect. 5 introduces the data that is analyzed, discusses the research question that is investigated here and presents some first empirical evidence. Moreover, this section also examines some methodological issues of relevance. In Sect. 6 the main empirical findings of this paper are reported and evaluated. Sect. 7 then finally concludes.

## Is there a dividend puzzle?

Miller and Modigliani (1961) have caused a controversial discussion in the field of financial economics with their idea that under certain circumstances the dividend policy of a corporation is irrelevant for the stock price of this firm. More specifically, they have argued that this is true when assuming that taxes do not exist, that there are perfect capital markets with rational investors, and that the firm’s investment policy is given. According to their theory higher dividend payments would simply lead to lower capital gains for equity investors and, as a consequence, the firm’s dividend policy should be described as irrelevant when investors do not prefer dividends to capital gains or vice versa. Accepting this point of view, there is a dividend puzzle because managers of many firms use resources to formulate a dividend policy. The idea that there is a puzzle is closely tied to the work of Black (1976). The existence of taxes may be of relevance at this point. However, it has been stressed that in at least some jurisdictions dividends are taxed more heavily than capital gains and that many firms still tend to pay dividends in spite of the tax disadvantages (see, for example, Black 1976; and Baker and Weigand 2015). In these countries the presence of taxes clearly makes the existence of dividend payouts even more “puzzling”. Although Bernheim (1991) has presented a theoretical model that could help to explain this phenomenon, there still are many open questions and the existence of taxes is generally not considered to be the main answer to the question why firms pay dividends. In any case, the empirical results that have been reported by Kalay and Michaely (2000) are very interesting in this context.

Agency theory is often seen to be a more promising solution to explain why firms pay dividends (see, amongst others, Baker et al. 2002; and Jiraporn and Chintrakarn 2009). Most importantly, Aivazian et al. (2003) have noted that dividend payments can force a firm to interact with its investors more frequently, because an outflow of funds through dividend distributions should cause a company to more often be in need to obtain capital from external sources in order to finance new investment projects. The process of raising new capital can help to reduce agency costs because the managers of the firm (as insiders) have to provide additional information to the public (the outsiders) to find investors (see, for example, Aivazian et al. 2003; and Basse and Reddemann 2011). While this explanation of the existence of dividend payments is very plausible, it has to be noted that obtaining new capital from external sources normally generates transaction costs. Consequently, it must be concluded that not all payout policy measures that reduce agency costs will make sense from an economic point of view.

In any case information asymmetries could play an important role in solving the dividend puzzle. As a matter of fact, according to the so-called dividend signaling hypothesis the managers of a firm (as insiders) could make changes to the volume of the dividends paid by a corporation to provide more information to its investors and the public (see, for example, Bhattacharya 1979; and Miller and Rock 1985). Consequently, the dividend policy followed by a firm should provide information about this business enterprise that up to now has not been available to outsiders. More specifically, the dividend signaling hypothesis predicts that dividends ought to be helpful forecasting corporate earnings (see, for instance, Goddard et al. 2006; and Basse and Reddemann 2011). This aspect will be of high importance later on.

However, dividend cuts in particular could also be quite problematic for the management of a firm. Balachandran et al. (2012), for example, have shown that investors generally react negatively to dividend reductions examining a very interesting data set from Australia. Moreover, Docking and Koch (2005) have argued convincingly that the market environment might also be of importance in this context. They have noted that the announcement of a dividend cut by a firm may lead to a significantly greater decrease to its stock price when market returns have been up and more volatile. In any case, some observers seem to think that financial markets could interpret dividend cuts or omissions as a clear sign for future problems. This should obviously be a particularly troublesome problem when the stock price would overreact to the new “information” about dividend payouts (see, for example, Ghosh and Woolridge 1989; and Lie 2005). As a matter of fact, Lintner (1956) has argued that because of the fears about an overreaction of stock prices to dividend cuts or omissions firms could try to prevent the occurrence of a situation in which the need for erratic adjustments to their dividend payouts emerges. Consequently, managers of the firms might have an incentive to only gradually increase their dividend payments in order to avoid the need for reductions to the volume of dividend distributions when facing problems. Given that dividends are paid from corporate earnings this would imply that managers should only announce increases to the volume of dividend payouts when it is highly likely that (of course absent a major crisis that could be used as “good” explanation) future earnings will allow the increased dividend payment under normal conditions. Phrased somewhat differently, the management of a firm does not want to be forced to cut or even omit dividend payments when there is no major crisis that could be used as explanation (or even as excuse) for the need to reduce dividend payments. This strategy which should help to make sure that dividend reductions remain to be an exceptional event is called dividend smoothing (see, amongst others, Bhattacharyya 2007; and Javakhadze et al. 2014).

According to Goddard et al. (2006), both, the dividend signaling and the dividend smoothing hypothesis, assume that a close relationship between corporate earnings and dividends should exist. In fact, Reddemann et al. (2010) have argued that dividend smoothing effectively is dividend signaling with precaution. From a very similar perspective, Karpavičius (2014) has proposed a very interesting interpretation of these two strategies that could create problems using the traditional tests of the dividend signaling hypothesis, which examine the relationship between dividends and the future performance of a firm. Goddard et al. (2006) have suggested a different way of testing for dividend smoothing or dividend signaling. Their approach clearly is of major importance for our empirical study and will be examined in more detail later on.

## European insurers and their dividend policy: what is already known?

There already have been some efforts to examine historical data in order to understand why many firms do pay dividends. In fact, several different empirical research strategies have been implemented to gain new insights and to tackle the dividend puzzle. The earlier empirical studies that examined dividend policy issues usually have employed techniques of cross-sectional regression analysis to examine dividend policy issues. However, many of the more recent studies meanwhile have focused on time series data. One reason for this new trend could be the work by Sarig (2004). He has argued that only the time series approach to empirical data analysis is capable to adequately describe the dynamics of corporate payout decisions. At this point, it is important to note that many of these newer empirical studies analyze the question whether dividend signaling or dividend smoothing are phenomena of economic relevance (see, for example, Goddard et al. 2006; and Basse and Reddemann 2011). Until recently most empirical studies of dividend policy issues have focused quite strongly on industrial companies (see, for example, Basse et al. 2014; and Basse 2019). This is probably no major surprise because it is also apparent that many of the earlier research papers clearly have focused on the theoretical explanations that could help to better understand the dividend policy of industrial firms. Obviously, there was less interest in the past to investigate this question from the perspective of financial services firms. In fact, in his seminal empirical study of dividend policy issues in the United States, Rozeff (1982) has not examined data from banks, insurance companies and all other regulated industries in order to focus on more unregulated industries.

However, there have been some relevant studies examining the financial services industry. These papers mainly report empirical evidence from North America. Mayne (1980), for example, has examined the dividend policy of banks in the United States. The results that have been reported in this study seem to imply that the size of a firm could have an influence on its dividend policy. This might be a consequence of the fact that larger banks tend to have better access to new external capital than smaller ones. As a result, the bigger financial institutions should be less dependent on internally generated funds. Moreover, Boldin and Leggett (1995) have reported empirical evidence that could be interpreted as supportive for the dividend signaling hypothesis examining data from the banking industry in the United States. In this study one very important point has been discussed in some detail that also is of some relevance for our paper. In fact, Boldin and Leggett (1995) have noted that higher dividend payouts can weaken the financial strength of a bank because the increased distribution of dividends to investors implies a smaller contribution to the capital of a firm. Meanwhile, there are also several additional empirical studies that analyze the dividend policy of the banking industry in the United States (see, for example, Dickens et al. 2003; and Theis and Dutta 2009). Moreover, Collins et al. (1996) have expanded the work of Rozeff (1982) by also examining banks and insurance companies. Interestingly, the results that have been reported in this paper are quite similar to the findings of Rozeff (1982). Collins et al. (1996) have argued that investors might believe that bank and insurance regulation could also help to effectively reduce agency costs. However, their study does not report clear evidence for a special role of regulatory constraints examining the dividend policy of banks and insurance companies in the United States. Additionally, there are some empirical studies that solely focus on the dividend policy of insurance companies in the United States (see, for example, Harrington 1981; and Casey et al. 2007). Puleo et al. (2009), for example, have reported that there seems to be no relationship between good corporate governance and dividend policy examining data from 55 US insurance companies. They have argued that this empirical finding could be a result of tight government regulations and have noted that further research is needed to understand the relationships among corporate governance mechanisms, regulation and dividend payouts in the financial services industry. Another important study analyzing data from North America seems to be Akhigbe et al. (1993). Employing an event study methodology and focusing on firms in the United States these authors have examined the stock price response of insurers and matched control samples of banks and industrial firms to dividend increases. They have reported a statistically significant positive response of the stock price in the insurance industry and have shown that the magnitude of this response is smaller for life insurers than for other types of insurance companies or industrial companies, but greater than that for firms from the banking industry. Akhigbe et al. (1993) have argued that this empirical finding could be a result of the relatively low level of capital maintained by life insurers.

Meanwhile, more and more empirical studies have also examined data from the European insurance industry to search for evidence indicating whether dividend signaling or dividend smoothing are of relevance here. Most importantly, Reddemann et al. (2010) have employed conventional Granger causality tests and multivariate techniques based on techniques of cointegration analysis to search for lead-lag-relationships between dividends and corporate earnings in this sector of the European economy. This study has examined quarterly data (Q1 1999 to Q4 2008) and has found no empirical evidence for dividend signaling. However, mixed results have been reported testing for dividend smoothing. More recently, Basse (2019) has reported additional empirical evidence examining this issue. He has argued that structural change could be of some importance in this context. In fact, this empirical study only analyzes quarterly data from the period Q1 2002 to Q1 2018 in order to avoid problems with structural change in the year 2001 and has reported clearer evidence for dividend smoothing in the European insurance industry than Reddemann et al. (2010). While Basse (2019) in general can be seen as an update of Reddemann et al. (2010), it only uses multivariate cointegration techniques to test for Granger causality because this approach is known to be able to cope with possibly relevant endogenity problems and also has a number of additional advantages. Moreover, Basse et al. (2011a) already have examined data from the Italian insurance industry. Their sample period is from Q1 1999 to Q4 2008 and they have also found empirical evidence for dividend smoothing and no support for the dividend signaling hypothesis using multivariate cointegration techniques to test for Granger causality among dividend payouts and corporate earnings. Furthermore, Jakubik and Teleu (2021) have examined daily data from 33 European insurance and reinsurance companies and using an event study methodology have shown that the drop of stock prices as a reaction to relevant negative information about dividends due to regulatory measures seems to have been quite limited.

## The recent crisis events and the Italian economy

It is still not absolutely clear how the so-called subprime crisis—a problem in a rather small segment of overall US financial markets—was able to cause a global financial crisis that hurt the world economy so badly (see Bullard et al. 2009; and Eichengreen et al. 2012). The European financial services industry might be of some relevance in this context (see, for example, Mizen 2008; and Noeth and Sengupta 2012). In fact, some European banks with substantial holdings of mortgage backed securities played an important role because these financial institutions had a direct exposure to the US real estate market (see, for example, Hellwig 2009; and Noeth and Sengupta 2012). Noeth and Sengupta (2012), for example, have argued convincingly that fears about a deterioration of the quality of bank balance sheets was at the heart of the financial woes in Europe. These concerns led to a significant worsening of the refinancing capabilities of banks in Europe. From this point on, at the latest, the problems also affected financial institutions that had no holdings of U.S. mortgage backed securities at all. In addition, the risk aversion of investors increased in general. More specifically, the US subprime crisis raised the awareness of market participants that there could also be neglected risks buying government bonds issued by less fiscally prudent countries that had introduced the Euro in the year 1999 or later. With regard to this issue it is important to note that Chang and Leblond (2015) have examined the behavior of fixed income investors before, during and after the European sovereign debt crisis and have documented significant changes in the perception of risks. Moreover, concerns about costly government bank bailout programmes started to emerge (see, amongst others, Basse et al. 2012; and Avino and Cotter 2014). From the perspective of at least some observers both factors might have helped to turn the US subprime debacle into a European sovereign debt crisis.

**Fig. 1:**
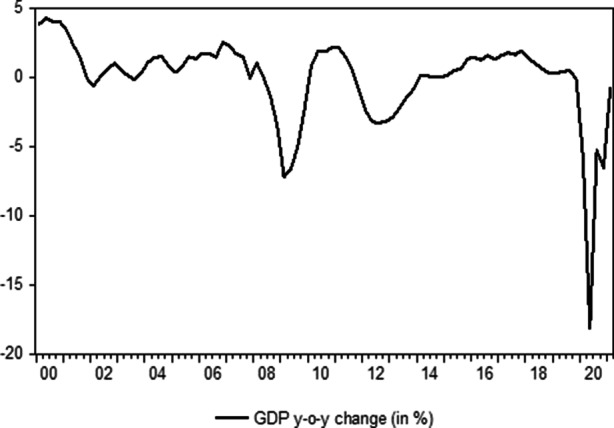
Italian GDP Q1 2000 to Q1 2021. (Source: Own representations based on data by ISTAT)

**Fig. 2:**
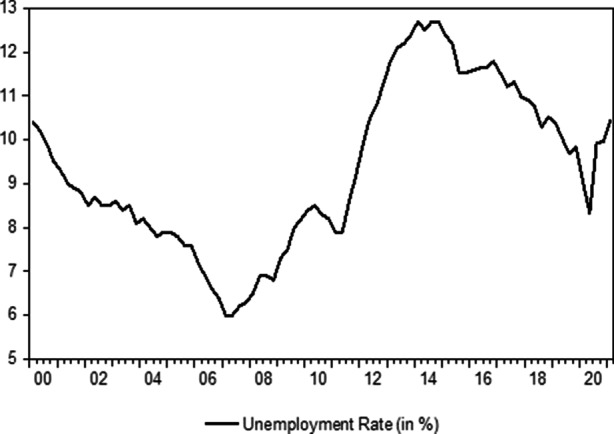
The employment situation in Italy. (Source: Own representations based on data by ISTAT)

The economic environment in the countries that were hit particularly hard by the European sovereign debt crisis was very different prior to the US subprime collapse. Filoso et al. (2021) have argued convincingly that real economic growth rates in Ireland, Greece and Spain were among the highest in the Eurozone before 2007 while the ones in Italy and Portugal were among the lowest. Italy has therefore already entered the crisis facing certain economic headwinds. In any case, the country has had to cope with some economic difficulties over the past 20 years. This can be clearly seen in the Figs. 1 and 2, which display the growth rates of the real gross domestic product (year-over-year change) and the unemployment rate in Italy (both time series are obtained from the Istituto Nazionale di Statistica (ISTAT)).

**Fig. 3:**
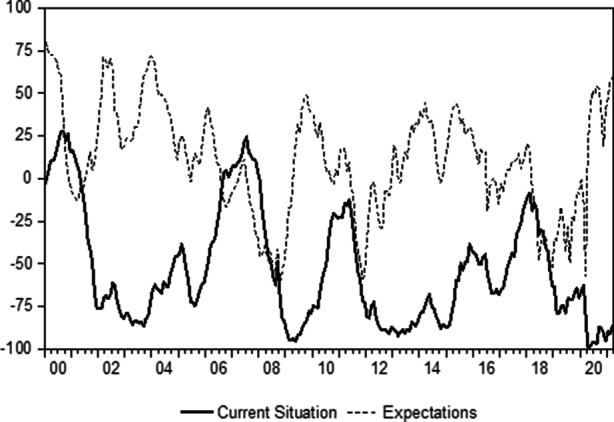
Economic sentiment indicators for Italy: A view from Germany. (Source: Own representations based on data by ISTAT Zentrum für Europäische Wirtschaftsforschung)

Without any doubt, the European sovereign debt crisis then brought about particularly significant challenges for the Mediterranean country. German institutional investors, for example, began to look more pessimistically at Italy’s economy again from 2012 onwards. Fig. 3 shows data from the German Zentrum für Europäische Wirtschaftsforschung for Italy. The sentiment indicators from this economic research institute are based on a monthly survey among portfolio managers, analysts and economists that are working for German financial services firms and are available for Italy and a number of other important countries (see, for example, Lux 2009; and Entorf et al. 2012). There are two time series—the forward-looking expectations of economic growth indicator (six months ahead) and the current situation index. The value that is reported measures the difference between the optimistic and pessimistic responses. Therefore, +100 is the maximum (only optimistic responses) and −100 is the minimum (only pessimistic responses). The data shows that the German investment professionals that responded to the questions of the Zentrum für Europäische Wirtschaftsforschung were just as critical of the current state of the Italian economy during the European sovereign debt crisis as they were during the subprime debacle. In the opinion of the survey participants, this period of weakness also persisted for a remarkably long time. In any case, after the sovereign debt default of Greece investors that held Italian government bonds started to have fears about sovereign credit risk and possibly even redenomination risk (see, for example, Sibbertsen et al. 2014; and Filoso et al. 2021). In this context it has to be noted, that redenomination risk is a special type of exchange rate risk (see, for example, Basse et al. 2018; and Tholl et al. 2020). It arises from the threat of the introduction of a new devaluing currency in a country after a possible exit from a monetary union. As a consequence, the government in Rome had to pay higher risk premia in order to generate demand for Italian sovereign bonds. Interest rates rose accordingly in the years 2010 and 2011, which in turn became a major problem for public finances and the Italian economy. Most importantly, the loss of confidence that is associated with the increase in sovereign credit risk and redenomination risk can become a major problem for the financial services industry of a country. This is evident, for example, from examining the experiences of the banking sector in Greece during the European sovereign debt crisis.

These turbulences certainly contributed to the decision of the Italian Prime Minister Silvio Berlusconi to announce his intention to resign. He stepped down in November 2011 and was succeeded by the former EU commissioner Mario Monti (see, for instance, Tholl et al. 2020; and Filoso et al. 2021). Filoso et al. (2021) have stressed that this change in the leadership of the country has helped to calm the turmoil on financial markets but have also pointed out that new problems among European banks (especially in Spain and Cyprus) caused fresh concerns. Investors again seemed to fear a collapse of the financial system in the European Monetary Union. Most importantly, Donadelli et al. (2020) have shown that rising political uncertainty has had clear implications for Italian asset prices in 2012 and onwards reporting empirical evidence based on a macroeconomic policy uncertainty index constructed using newspaper articles. In this difficult situation economic policy makers in Rome faced big challenges. Succeeding Mario Monti, the country had three other heads of government between the years 2013 and 2018, with Enrico Letta, Matteo Renzi and Paolo Gentiloni, before Giuseppe Conte became the new Italian Prime Minister, being an independent leader of a coalition government which was formed after the election in 2018. Later on, there were additional changes to the government and Mario Draghi, the former president of the European Central Bank, emerged as Prime Minister of Italy following Giuseppe Conte in February 2021 (see, amongst others, Garzia and Karremans 2021; and Newell 2021). After only a rather short time in this role Mario Draghi resigned as Prime Minister in 2022. Meanwhile elections took place again resulting in a new government that is now lead by Giorgia Meloni.

Afonso et al. (2018) have argued convincingly that the European Central Bank announced new monetary policy measures in August 2012 to improve the liquidity situation in financial markets. As a matter of fact, many interested observers of European monetary policy meanwhile seem to believe that the now famous speech of Mario Draghi (“whatever it takes”) has helped to significantly reduce fears about sovereign credit risk and especially also about redenomination risk among fixed income investors (see, for instance, Klose and Weigert 2014; and Tholl et al. 2020). These measures therefore also helped to improve the economic environment in countries like Portugal, Spain and Italy. As already noted, the elections in March 2018 caused new political turbulences in Rome (see, amongst others, Cozzolino 2020; and Tholl et al. 2020). Tholl et al. (2020), for example, have argued convincingly that the formation of the new government by the parties Lega (League) and Movimento Cinque Stelle (M5S—Five Star Movement) in 2018 led to at least temporarily higher risk premia for Italian government bonds. Meanwhile, the economic effects of the Covid-19 pandemic have come into focus (see, for instance, Giammetti et al. 2020; and Tholl et al. 2020). This crisis clearly has hit Italy very hard. As already noted, the regulatory authorities in Italy reacted to these problems by trying to limit the ability of insurance companies to pay dividends (see, most importantly, Jakubik and Teleu 2021).

**Fig. 4:**
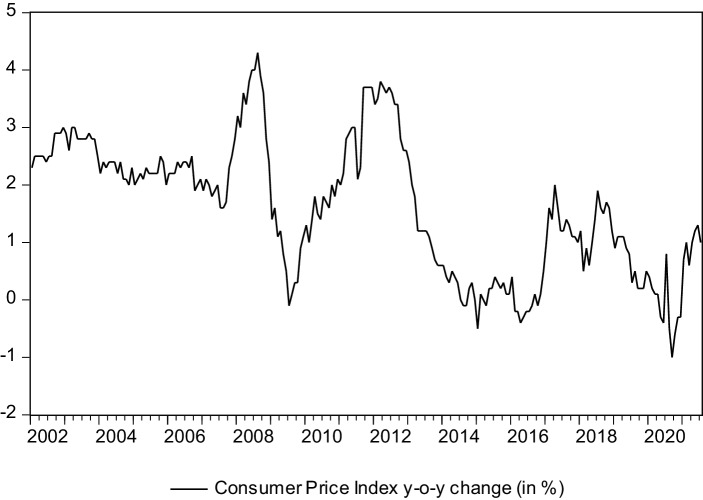
Inflation in Italy. (Source: Own representations based on data by ISTAT)

The Covid-19 pandemic recently also has caused some deflationary tendencies in Italy. Apart from this short phase, inflation rates (consumer prices—the data set again is obtained from the Istituto Nazionale di Statistica (ISTAT)) in Italy were quite stable during the period under review and, overall, rather low. Indeed, Fig. 4 shows that values above 4.0% have not been realized in the period under observation here (that means, as will be explained later, since 2009 and before the second half of 2021). However, it has to be noted that the inflationary environment has changed significantly in recent times. Sharp rises to consumer prices are observable in the European Monetary Union as a whole—and also in Italy. In fact, increases of the consumer price index above the 10% year-over-year mark can now be witnessed in many countries in Europe. Italy is no exception in this regard and in the meantime the European Central Bank was forced to hike interest rates in order to combat higher inflation rates. That said, this has clearly been a phenomenon of the very recent past. The changes that the European Central Bank meanwhile has made to the stance its monetary policy may also cause additional fears about redenomination risk because higher bond yields could be quite problematic for the more indebted member countries of the monetary union. Without any doubt, financing costs for governments have increased as a result of the interest rate hikes that recently have been implemented by the European Central Bank. As a consequence, at least some investors have started to worry about the issue of debt sustainability again. Of course, these concerns primarily affect the countries in the European periphery—in other words, the usual suspects.

It has been argued that inflation might have an effect on tests for dividend signaling or dividend smoothing using the techniques of time series analysis (see, most importantly, Basse and Reddemann 2011). Therefore Basse et al. (2011a) have include a measure of the macroeconomic price level in Italy analyzing the dividend policy of insurance companies in this country. Given the data that is shown in Fig. 4 this should not be required here. In fact, the rather short sample that is examined in the present study—which is a result of data limitations—makes the use of such an approach seem undesirable. As will be discussed later on in more detail, we employ an approach that is based on the technique of vectorautoregressions which has been suggested by Sims (1980). This type of model requires the estimation of many parameters when a number of variables and more than just one or two time lags are considered to analyze the relationships among the time series that are under investigation. This can cause problems with the degrees of freedom. Therefore, given that the inflation rate in Italy has been low and rather stable since 2009 (and before the recent increase which is just outside our observation period) we have decided to not include a measure of inflation in our empirical models.

## Data and some methodological issues

It has been suggested that European banks (see, for example, Basse et al. 2014; and Cohen and Scatigna 2016) and European insurance companies (see, for instance, Reddemann et al. 2010; and Jakubik and Teleu 2021) could reduce their dividend payouts in a crisis in order to strengthen their capital base. Doing so might indeed be helpful to cope with some of the difficulties that arise in a crisis. However, omitting or cutting dividends could also be a problem. In fact, it has been argued that the managers of firms pay dividends in order to meet the demands and needs of their investors (see, for instance, Ferris et al. 2009; and Baker and Jabbouri 2016). Therefore, reducing or omitting dividends in order to increase the ability to act in the event of future complications might be problematic when the management of a firm follows the strategy of dividend signaling, because investors then might think that this is a clear indication for major problems ahead. Stated somewhat differently, market participants could be of the opinion that the implemented dividend cuts or omissions are not helping to solve problems but could be a sign that the management of a firm foresees new trouble ahead. As already noted, this would be particularly troublesome if stock prices overreacted to such signals. Moreover, the idea to lower dividends in order to prepare for future difficulties could also be problematic for firms that smooth dividends because in this case investors in general seem to prefer stable dividend payouts. Thus, firms that engage in dividend signaling or dividend smoothing should therefore be very cautious when making changes to their payout policy. These companies should at least communicate their plans very clearly when they announce lower dividends in order to strengthen their capital base preemptively preparing for problems that might possibly occur in the coming quarters. Moreover, firms that follow this strategy with regard to their payout policy could obtain a special benefit from the possibility of being able to present good reasons for dividend cuts or omissions that are resulting from regulation. In any case, cutting or even omitting dividend payments in order to improve the capital base of a corporation may be easier for firms that do not practice dividend signaling or dividend smoothing because the investors in these companies seem to care less about changes to the volume of dividend payouts.

Therefore, this empirical study tests for dividend smoothing or dividend signaling in the Italian insurance industry. More specifically, we follow the approach that has been suggested by Goddard et al. (2006). As already noted, this empirical research strategy is based on the concept of Granger causality (see, most importantly, Granger 1969, 2003) which will be discussed in more detail later on. According to the dividend signaling hypothesis dividend payouts ought to lead corporate earnings and according to the dividend smoothing hypothesis corporate earnings should lead dividends. Finding Granger causality that runs from dividends to corporate earnings therefore would be supportive for the dividend signaling hypothesis while empirical evidence for corporate earnings Granger causing dividend payments would point in the direction that the management of a firm engages in dividend smoothing (see, most importantly, Goddard et al. 2006).

As previously mentioned, this paper analyzes data from the Italian insurance industry. In fact, this empirical study follows Basse et al. (2011a). We also examine quarterly data. However, given that the Milan Stock Exchange Insurance Companies Index analyzed by Basse et al. (2011) was discontinued in June 2009, our study focuses on the Italian FTSE All-Share Insurance Index. More specifically, we examine the dividend per index share (DPS) and the earnings per index share before extraordinary items (EPS) time series. Additionally, we also look at the stock price of the FTSE All-Share Insurance Index (PRICE). All stock market data is taken from Bloomberg. The dividend data for the FTSE All-Share Insurance Index is available since the year 2009 (to be more precise since Q2 2009). The sample under investigation here therefore is Q2 2009 to Q1 2021. Thus, there are just enough data points to perform the test procedure that is employed here. As already noted the data constraints are the reason why we do not include a measure of inflation in our empirical models. However, the rather short sample has one advantage because we focus on the experiences after the Global Financial Crisis. This should help to minimize problems due to structural change. Tallman and Zaman (2020), for example, recently have suggested that it is one possible empirical research strategy to shorten the sample that is analyzed to cope with the difficulties that result from structural change. As a matter of fact, it is a quite common procedure in applied econometrics to examine shorter samples in order to avoid problems that are caused by structural change (see, most importantly, Walsh and Wilcox 1995).

As already noted, the empirical research strategy that is employed in this study is based on the concept of Granger causality. A time series *Y* is Granger causing the variable *X* if it can help forecast the time series *X*. More specifically, the variable is said to not Granger cause the time series if for all $n> 0$:

1 $$F\left(Y_{t+n}|\Omega _{t}\right)=F\left(Y_{t+n}|\Omega _{t}-X_{t}\right).$$

In Eq. 1*F* denotes the conditional distribution and Ω_*t*_ describes all information that might be of relevance.

The three time series under investigation here are possibly non-stationary variables. Therefore, we employ the procedure suggested by Toda and Yamamoto (1995) in order to test for Granger causality. As already noted, this approach is based on the concept of vector autoregressions (see, most importantly, Sims 1980) and has a number of advantages. First of all, it does not require much pretesting. Additionally the technique which has been developed by Toda and Yamamoto (1995) can be used to analyze data sets that included both, stationary and non-stationary variables, in a very elegant way. Moreover, given that the approach is based on the concept of vector autoregression this test procedure is able to adequately model the complex dynamic interaction among the time series under investigation. In Eq. 2*Y*_*t*_ is a vector of $(n\times 1)$ endogenous variables, *A*_*i*_ are $(n\times n)$ coefficient matrices and *ε*_*t*_ is a disturbance term:

2 $$Y_{t}=A_{1}\cdot Y_{t-1}+A_{2}\cdot Y_{t-2}+\ldots +A_{n}Y_{t-n}+\varepsilon _{t}.$$

Moreover, it is possible to add a $(n\times 1)$ vector of constants or seasonal dummy variables to this model. Toda and Yamamoto (1995) have proposed to estimate a vector autoregression in levels even when examining non-stationary variables. They have suggested to include surplus time lags to the model to ensure that the test statistic is asymptotically chi-square distributed when searching for Granger causality. More specifically, the model which is shown in Eq. 3 considers *p* time lags and is extened by *m* additional surplus time lags to perform modified Wald tests to search for Granger causality. Here *m* is the highest order of integration of any variable that is considered in the model and *p* is the optimal number of time lags for the vector autoregression:

3 $$Y_{t}=A_{1}\cdot Y_{t-1}+A_{2}\cdot Y_{t-2}+\ldots +A_{p}Y_{t-p}+A_{p+m}Y_{t-\left(p+m\right)}+\varepsilon _{t}.$$

Again, a constant or seasonal dummy variables might be added to this model. This will be necessary given the strong seasonal pattern in the dividend time series. As already noted, finding Granger causality that runs from dividends to corporate earnings would be supportive for the dividend signaling hypothesis (see, most importantly, Goddard et al. 2006). Likewise, empirical evidence that earnings Granger cause dividend payments would speak for the dividend smoothing hypothesis. According to the results of the unit root tests reported in the Tables 1, 2 and 3, two of the time series under investigation (namely stock prices and the dividend time series) are non-stationary variables integrated of order one. The third variable corporate earnings seems to be stationary. The critical values for the ADF tests are taken from MacKinnon (1996). These interesting empirical findings make a strong case for the use of the test procedure that has been suggested by Toda and Yamamoto (1995) because a mixture of stationary and non-stationary variables is examined here. Given that we estimate bivariate models and that only one time series seems to be stationary while the other variables under investigation all are considered to be integrated of order one, the value of *m* always is one in all models examined here.

**Table 1:** Unit root tests dividend per index share. (Source: Own representations)

Null Hypothesis: Time series has a unit root		
*Exogenous: Constant*		
	Data	Data
	In levels	In first differences
ADF test statistic	−2.2712	−30.8131
5% critical value	−2.9297	−2.9297

**Table 2:** Unit root tests earnings per index share. (Source: Own representations)

Null Hypothesis: Time series has a unit root		
*Exogenous: Constant*		
	Data	Data
	In levels	In first differences
ADF test statistic	−4.3885	−5.8624
5% critical value	−2.9266	−2.9266

**Table 3:** Unit root tests stock prices (Source: Own representations)

Null Hypothesis: Time series has a unit root		
*Exogenous: Constant*		
	Data	Data
	In levels	In first differences
ADF test statistic	−2.6167	−6.6444
5% critical value	−2.9252	−2.9266

## Empirical analysis

In order to determine the value of *p* the Akaike information criterion is used. Analyzing the relationship between dividends and corporate earnings *p* equals four according to this approach. As a consequence, Eq. 3 is estimated with $p=4$ and $m=1$ in this case. As already noted, the technique that has been suggested by Toda and Yamamoto (1995) ensures that the test statistic for the augmented Wald tests is asymptotically chi-square distributed. The results reported in Table 4 seem to indicate that the hypothesis of Granger causality not running from dividend payments to corporate earnings can be rejected with a *p*-value of 0.0025. However, there is no evidence that the earnings per index share time series Granger causes the dividend per index share time series. As a matter of fact, the hypothesis of no Granger causality cannot be rejected in this case. A look at the details here shows a *p*-value of 0.9200. Thus, we have documented the existence of unidirectional Granger causality running from dividend payments to corporate earnings. Consequently, there is strong empirical evidence for dividend signaling in the Italian insurance industry examining data from Q2 2002 to Q1 2021 following the approach that has been introduced by Goddard et al. (2006).

**Table 4:** Granger causality tests examining dividends and earnings per share. (Source: Own representations)

TY Granger Causality Tests			
*Dependent variable: DPS*			
Excluded	Chi-sq	Df	Prob
EPS	0.931798	4	0.9200
*Dependent variable: EPS*			
Excluded	Chi-sq	Df	Prob
DPS	16.44791	4	0.0025

Therefore, accepting the point of view that the managers of firms decide to pay dividends in order to meet the needs and demands of their investors (see, for example, Ferris et al. 2009; and Baker and Jabbouri 2016), cutting or omitting dividends in order to strengthen the capital base of an insurance company in Italy could be problematic because the stockholders seem to want to obtain information about future profits from the dividend payments of the insurers. This has been highlighted very clearly by Reddemann et al. (2010). Phrased somewhat differently, empirical evidence for dividend signaling could be interpreted as a sign that the owners of these business enterprises want to examine the payouts in order to draw conclusions about the private information available to the managers regarding the future economic situation of a company. Therefore, dividend cuts or omissions that are implemented to improve the ability of the firms to better cope with future problems could be misinterpreted by investors as a sign for the knowledge of the management about even greater difficulties in the coming periods. Jakubik and Teleu (2021), for example, have argued convincingly that this would be very problematic when stock prices overreact to dividend cuts or omissions. Following this reasoning the insurance companies that decide to cut or even omit dividends in order to strengthen their capital base and that thereby try to limit as much as possible any potential need to issue new equity in times of crisis—a situation in which stock prices tend to be low—should at least communicate very clearly what they are doing and why they are making these changes to their payout policy.

However, the results that are reported in Table 4 should be interpreted with great caution only. In fact, insurance companies in Italy have decided to cut dividends after the global financial crisis. Cohen and Scatigna (2016) have shown that such a behavior was in general observable in the European financial services industry after the crisis. Lower dividend payouts helped financial services firms to accumulate additional capital by retaining earnings. This procedure to be observed in the “real world” most likely was based on the idea that the firms wanted to shield themselves from possible future problems with the implementation of this strategy. Again, assuming that managers want to satisfy the demands of their investors this should be no problem. Moreover, the recent crisis events have also led the regulators of the financial services industry in many regions of the world to make changes to capital requirements (see, for example, Benczur et al. 2017; and Fidrmuc and Lind 2020). Consequently, the quantity of capital that financial services firms are forced to set aside to tackle possible unexpected future losses has increased in recent times. Examining bank stock returns in 52 counties Igan et al. (2022), for instance, have shown that capital surcharges on systemically important financial institutions seem to affect market expectations about future profits in a negative way. This empirical finding should be of some importance for bank regulators and investors. In this context it has to be noted that Meier et al. (2021) have given a wide-ranging overview of the effects of the global financial crisis and the European sovereign debt crisis on the regulation of banks and insurance companies. Moreover, there are interesting discussions about regulatory restrictions on dividend payouts in the financial services industry that are of relevance for our empirical study (see, amongst others, Lepetit et al. 2017; and Juelsrud and Nenov 2020). Most importantly, Jakubik and Teleu (2021) have shown that this aspect is of high importance for the European and Italian insurance industry. This issue will be considered in more detail in the following discussion. In fact, it might be helpful for managers in the banking and insurance industry to be able to argue that adjustments to dividend payouts are made due to regulatory requirements. Furthermore, it has to be pointed out that Nguyen et al. (2020) have provided evidence for the fact that financial institutions at least try to take adequate measures to meet higher capital requirements. Thus, the empirical findings reported above could simply be the consequence of a foresighted behavior of the managers of the companies that is in the interest of their shareholders. Both interpretations would somehow be compatible with the idea that dividend payouts provide information about future developments (which, as already noted, is called dividend signaling in the literature). The big difference is that preparations of the management for a generally more difficult economic environment are not (or at least less strongly) to be seen as a reaction to firm-specific information.

At this point it might be a good idea to also examine stock prices (see, most importantly, Goddard et al. 2006). More specifically, we test for Granger causality (respectively no Granger causality) among dividends payouts and stock prices. Again, the procedure that has been suggested by Toda and Yamamoto (1995) is used. Once more *p* is determined using the Akaike information criterion. The obtained value for this parameter is again 4. The results of the test are reported in Table 5.

**Table 5:** Granger causality tests examining dividends and stock prices. (Source: Own representations)

TY Granger Causality Tests			
*Dependent variable: DPS*			
Excluded	Chi-sq	Df	Prob
PRICE	10.45468	4	0.0334
*Dependent variable: PRICE*			
Excluded	Chi-sq	Df	Prob
DPS	1.727832	4	0.7857

While the hypothesis that dividends do not Granger cause stock prices cannot be rejected, there is empirical evidence for Granger causality running from stock prices to dividends (with a *p*-value of 0.0334). Consequently, the empirical findings reported in this study seem to imply that there is uni-directional Granger causality and that stock prices ought to be helpful trying to forecast future dividend payments. Phrased somewhat differently, current dividend payouts do not provide relevant information about future changes to stock prices, but stock price movements today help to predict changes to dividend distributions examining quarterly data. This finding is interesting because it is compatible with the predictions of the dividend discount model (see, for example, Charteris and Chipunza 2020; and Basse et al. 2021). In any case, the empirical evidence presented in this study does not suggest any major risks that the stock prices considered here will react too strongly to adjustments in dividend payments. However, it might still be a good idea for firms to communicate clearly and in an unambiguous way why dividend payments have been lowered when these adjustments are made to have greater financial flexibility in the future. Moreover, in order to explain the empirical findings that are reported in Table 5, it could also be argued that the recommendations of the European and Italian insurance regulators to at least exercise great caution when paying dividends in the year 2020 (or even refrain from dividend distributions) might play a role. In fact, insurance companies may have been able to use the recommendations of the regulators as some kind of “excuse” for their dividend reductions. However, the results displayed in Table 5 should certainly not be overinterpreted. This could be due to the short period of time that is examined here. As already discussed, the use of a data set consisting of a limited number of observations has both, advantages and disadvantages. Most importantly, we focus on a single episode in economic history, but one that—as a major crisis—is likely to have a very special importance. Thus, we examine the behavior of the firms under observation here during precisely this period. Among other things, this minimizes potential problems with structural breaks. In addition, the behavior of the companies in this crisis could be of particular interest. Consequently, our approach is in some respects similar to the event study methodology. However, in order to be able to reach more general conclusions about the dividend policy of the Italian insurance industry, it would certainly be helpful to examine a longer period of time, which is currently (as discussed) complicated because the lack of data. In the future this problem will, of course, be solved by itself due to the passing of time. In any case, it seems to be clear that further empirical evidence is needed with regard to this question. As a matter of fact, it could also be a good idea to examine data from individual Italian insurance companies in order to gain additional insights.

## Conclusion

Based on the approach that has been suggested by Goddard et al. (2006), we have found clear evidence for dividend signaling examining data from the Italian insurance industry after the Global Financial Crisis. Assuming that there is a reason for dividend payouts and that the managers of firms decide to pay dividends in order to meet the needs and demands of their investors (see, for example, Ferris et al. 2009; and Baker and Jabbouri 2016) this might be a problem because the empirical findings reported above could imply that the stockholders of the insurance companies in Italy want to obtain information about future profits from dividend payments. Therefore, dividend cuts or omissions that are implemented by the management in order to strengthen the capital base of the insurers and that might indeed help to better cope with future problems could be misinterpreted as clear sign for key decision makers of the firm anticipating greater difficulties in the coming periods. As a consequence, new difficulties could arise. This would especially be true for markets with tendencies of overreactions of stock prices as a result to dividend cuts or omissions. However, caution must be exercised in interpreting the results presented here. As a matter of fact, Italian insurers have decided to cut dividends after the global financial crisis and most likely wanted to shield themselves from future problems with the implementation of this strategy. Thus, the empirical findings reported above could simply be the consequence of a foresighted behavior of the managers of the companies. Both interpretations would somehow be compatible with the general idea behind dividend signaling (namely that dividends provide relevant information about future developments). In addition, the relevant regulatory authorities have attempted to limit the ability of insurance companies to pay dividends. This, of course, also does play a role here. In fact, the recommendations of the regulators with regard to dividend payouts might help to explain why there is no empirical evidence that current dividend payouts do provide relevant information about future changes to stock prices analyzing the data examined here. This finding clearly speaks against the hypothesis that there is an overreaction of stock prices to dividend cuts. It could be argued that the regulators’ requests have given companies a kind of “excuse” for dividend adjustments. However, the results of our empirical investigations should also not be overinterpreted. Indeed, the period considered here is quite short. That said, the lessons learned during this period probably have a very special relevance because of the magnitude of this crisis. Additional evidence examining this important issue is clearly needed. In any case, it seems appropriate for a company’s management to communicate well why dividends have been adjusted when these adjustments to the payout policy of a firm have been made to improve the ability of this entity to address problems that possibly could arise in the future. In this situation, the changes to the dividend payouts are no sign for impending new difficulties, but could even help to cope with upcoming challenges.
